# Genome-wide identification, characterization of the MADS-box gene family in Chinese jujube and their involvement in flower development

**DOI:** 10.1038/s41598-017-01159-8

**Published:** 2017-04-21

**Authors:** Liman Zhang, Jin Zhao, Chunfang Feng, Mengjun Liu, Jiurui Wang, Yafei Hu

**Affiliations:** 1 College of Life Science, Hebei Agricultural University, Baoding, 071000 China; 2 College of Forestry, Hebei Agricultural University, Baoding, 071000 China; 3 Research Center of Chinese Jujube, Hebei Agricultural University, Baoding, 071000 China; 4 BGI-Shenzhen, Shenzhen, 518083 China

## Abstract

MADS-box genes encode transcription factors that are involved in plant development control (particularly in floral organogenesis) and signal transduction pathways, though a comprehensive analysis of MADS-box family proteins in Chinese jujube (*Ziziphus jujuba* Mill.) is still missing. Here, we report a genome-wide analysis of the MADS-box gene family in Chinese jujube. Based on phylogenetic analyses, 52 jujube MADS-box genes were classified into 25 MIKC^C^-type, 3 MIKC^*^-type, 16 Mα, 5 Mβ and 3 Mγ genes. 37 genes were randomly distributed across all 12 putative chromosomes. We found that the type II genes are more complex than the type I genes and that tandem duplications have occurred in three groups of MADS-box genes. Meanwhile, some gene pairs in the same clade displayed similar or distinct expression profiles, suggesting possible functional redundancy or divergence. MIKC^C^-type genes exhibited typical temporal and spatial expression patterns in the four whorls of floral tissues. The expressions of B, C/D and E-type genes were significantly suppressed in phyllody as compared to flower, providing valuable evidence for their involvement in flower development. This study is the first comprehensive analysis of the MADS-box family in jujube, and provides valuable information for elucidating molecular regulation mechanism of jujube flower development.

## Introduction

MADS-box genes are transcription factors that play a significant role in plant development, especially in determining floral organ identities. Floral organ identity genes have traditionally been subdivided into five different classes (class A, B, C, D, and E genes) that provide five different “homeotic functions”, with A-class genes specifying sepals, A + B + E specifying petals, B + C + E specifying stamens, C + E specifying carpels, and D specifying ovules^1–3^.

Phylogenetically, MADS-box genes have been divided into two types, type I and type II. The type I genes have, in turn, been divided into three phylogenetic groups (Mα, Mβ and Mγ), whereas the type II genes have been further divided into MIKC^C^ and MIKC*-type genes based on both the different lengths of their encoded K-domains and on phylogenetic standards^4–6^. In angiosperms, MIKC^C^-type genes were further subdivided into 12 clades^7^.

Structurally, MADS-box genes possess a conserved DNA-binding domain, which is defined by a highly conserved 60-amino-acid sequence that is involved in binding to DNA based on a consensus CC(A/T)_6_GG (also known as the CArG box) sequence^8–10^. In addition to the highly conserved MADS domain, members of the type II lineage contain three additional domains (from N- to C-terminus): the Intervening (I) domain, the Keratin-like (K) domain and the C-terminal (C) region^11–14^. The K domain is the second most-conserved region and is responsible for dimerization via a coiled-coil structure, while the less conserved I domain may contribute to DNA-binding specificity and dimerization. The C-terminal region is highly variable, and it has been shown to function in protein complex formation and transcriptional activation^15–17^.

Chinese jujube (*Ziziphus jujuba* Mill.) is one of the most economically important fruit trees in China. Compared to other fruit trees, this tree has some unique reproductive features such as a fast flowering time (of approximately 7 days), a short juvenile phase and first-year fruiting ability. Flower bud differentiation and fruiting occur in the same year, and there has been no flower bud dormancy reported for the Chinese jujube. Hence, a genome-wide analysis of the jujube MADS-box gene family may be useful for revealing the unique flowering mechanism of this species at the molecular level.

In this study, we identified 52 MADS-box genes in the jujube genome, analyzed their phylogenetic relationships and gene structures, and predicted their chromosomal localization. Additionally, to study the role of these genes in jujube flower development, we used semi-quantitative RT-PCR and real-time quantitative RT-PCR (qRT-PCR) to determine expression profiles.

## Results

### Identification and classification of MADS-box genes in the Chinese jujube

After the screening process, a total of 52 non-redundant MADS-box proteins were identified and serially named as *ZjMADS1* through *ZjMADS52* (Table 1). The CDS length of the jujube MADS-box genes ranged from 387 bp to 1053 bp; the encoded proteins ranged from 128 to 350 amino acids (aa) in length (with an average of 235.92 aa), had a predicted molecular mass of 14.60–40.24 KDa, and protein pIs ranged from 4.34 to 9.91.

**Table 1:** The information of MADS gene family in Chinese jujube.

Gene name	NCBI Reference Sequence	Introduction	Chromosomes	Position	ORF (bp)	Size (aa)	MW(KD)	PI	Types	Group	Exon number
*ZjMADS1*	XM_016024542.1	MADS-box protein FBP24-like (LOC107416088)	Chr4	10183943–10184983−	1041	346	39642.7	7.66	Type I	Mβ	1
*ZjMADS2*	XM_016032882.1	floral homeotic protein AGAMOUS-like (LOC107423340)	Chr7	24407258–24408310−	1053	350	40235.8	8.59	Type I	Mβ	1
*ZjMADS3*	XM_016039896.1	agamous-like MADS-box protein AGL80 (LOC107429240)	Chr1	30739401–30740000+	564	187	21886.0	9.91	Type I	Mγ	1
*ZjMADS4*	XM_016021407.1	agamous-like MADS-box protein AGL80 (LOC107413453)	Chr3	8091417–8092190−	696	231	26631.7	9.55	Type I	Mγ	2
*ZjMADS5*	XM_016019321.1	agamous-like MADS-box protein AGL80 (LOC107411686)	Chr2	19143165–19143993−	735	244	27646.7	9.38	Type I	Mγ	1
*ZjMADS6*	XM_016026865.1	agamous-like MADS-box protein AGL103 (LOC107418187)	Chr1	10568363–10569199+	837	278	30843.6	4.40	Type I	Mβ	1
*ZjMADS7*	XM_016021296.1	agamous-like MADS-box protein AGL62 (LOC107413360)	Chr1	6306218–6306929+	642	213	24101.9	8.57	Type I	Mα	1
*ZjMADS8*	XM_016021288.1	agamous-like MADS-box protein AGL62 (LOC107413352)	Chr1	6297324–6298037+	714	237	26834.7	7.26	Type I	Mα	1
*ZjMADS9*	XM_016010897.1	agamous-like MADS-box protein AGL62 (LOC107403968)	unplaced genomic scaffold, ZizJuj_1.1 scaffold1156	24359–25057+	699	232	26103.9	9.34	Type I	Mα	1
*ZjMADS10*	XM_016010899.1	agamous-like MADS-box protein AGL62 (LOC107403970)	unplaced genomic scaffold, ZizJuj_1.1 scaffold1156	36825–37560+	699	232	26078.9	9.35	Type I	Mα	1
*ZjMADS11*	XM_016010903.1	agamous-like MADS-box protein AGL62 (LOC107403974)	unplaced genomic scaffold, ZizJuj_1.1 scaffold1156	65817–66688+	774	257	28356.5	9.58	Type I	Mα	1
*ZjMADS12*	XM_016010900.1	agamous-like MADS-box protein AGL62 (LOC107403971)	unplaced genomic scaffold, ZizJuj_1.1 scaffold1156	51228–51926+	699	232	25984.8	9.35	Type I	Mα	1
*ZjMADS13*	XM_016034931.1	agamous-like MADS-box protein AGL29 (LOC107425004), transcript variant X6	Chr8	10748903–10751975+	594	197	21432.4	9.02	Type I	Mα	1
*ZjMADS14*	XM_016021687.1	agamous-like MADS-box protein AGL29 (LOC107413672)	Chr1	6310096–6311773+	503	167	18434.1	9.55	Type I	Mα	1
*ZjMADS15*	XM_016015134.1	agamous-like MADS-box protein AGL29 (LOC107407822)	unplaced genomic scaffold, ZizJuj_1.1 add_scaffold288	3900–5826+	437	145	16296.3	5.27	Type I	Mα	1
*ZjMADS16*	XM_016043741.1	uncharacterized LOC107432568 (LOC107432568)	Chr12	8429463–8430775−	969	322	35425.4	5.74	Type I	Mα	1
*ZjMADS17*	XM_016012251.1	floral homeotic protein AGAMOUS-like (LOC107405224)	unplaced genomic scaffold, ZizJuj_1.1 add_scaffold2771	30556–31170+	615	204	22651.6	7.19	Type I	Mα	1
*ZjMADS18*	XM_016044201.1	transcription factor of morphogenesis MCM1-like (LOC107432968)	Chr12	13354391–13355152−	762	253	27621.5	5.09	Type I	Mα	1
*ZjMADS19*	XM_016046367.1	agamous-like MADS-box protein AGL61 (LOC107434868)	Chr1	36630630–36631220−	591	196	21567.6	8.73	Type I	Mα	1
*ZjMADS20*	XM_016039429.1	agamous-like MADS-box protein AGL61 (LOC107428834)	Chr1	30796422–30797094+	579	192	21428.4	9.25	Type I	Mα	2
*ZjMADS21*	XM_016010684.1	agamous-like MADS-box protein AGL62 (LOC107403770)	unplaced genomic scaffold, ZizJuj_1.1 scaffold1100	45304–46304+	453	150	16683.0	9.46	Type I	Mα	1
*ZjMADS22*	XM_016044983.1	agamous-like MADS-box protein AGL104	unplaced genomic scaffold, ZizJuj_1.1 add_scaffold2747	435984–438520+	1035	344	39458.3	5.08	Type II	MIKC*	11
*ZjMADS23*	XM_016035388.1	agamous-like MADS-box protein AGL66 (LOC107425386)	Chr8	14985824–14987965−	711	236	27067.7	6.10	Type II	MIKC*	9
*ZjMADS24*					721	240	27857.6	6.98	Type II	AP1	4
*ZjMADS25*	XM_016039573.1	agamous-like MADS-box protein AGL30 (LOC107428964), transcript variant X3	Chr10	10819991–10826125+	1035	344	38738.7	6.80	Type II	MIKC*	10
*ZjMADS26*	XM_016044922.1	MADS-box transcription factor 14-like (LOC107433624),	unplaced genomic scaffold, ZizJuj_1.1 add_scaffold2825	246193–262937+	672	223	25076.3	7.06	Type II	FIC	7
*ZjMADS27*	XM_016038819.1	truncated transcription factor CAULIFLOWER A-like (LOC107428302)	Chr10	2883544–2902582−	648	215	25114.3	8.85	Type II	FIC	7
*ZjMADS28*	XM_016043834.1	MADS-box protein SOC1 (LOC107432650)	Chr12	9493439–9500235−	777	258	29601.4	9.74	Type II	SOC	6
*ZjMADS29*	XM_016040950.1	agamous-like MADS-box protein AGL19 (LOC107430150)	Chr11	3615968–3628803+	684	227	26483.9	9.57	Type II	SOC	9
*ZjMADS30*	XM_016039731.1	MADS-box protein CMB1-like (LOC107429081)transcript variant X1	Chr10	13870228–13875103−	735	244	27878.6	7.63	Type II	SEP	8
*ZjMADS31*	XM_016039725.1	truncated transcription factor CAULIFLOWER A (LOC107429076)	Chr10	13771665–13776993−	738	245	28544.7	8.65	Type II	AP1/FUL	7
*ZjMADS32*	XM_016020941.1	floral homeotic protein AGAMOUS (LOC107413065), transcript variant X3	Chr1	6013152–6023107−	735	244	27797.5	9.55	Type II	AG	7
*ZjMADS33*	XM_016043858.1	agamous-like MADS-box protein AGL6 (LOC107432672)	Chr12	9481942–9487212+	750	249	28308.2	9.38	Type II	AGL6	8
*ZjMADS34*	XM_016030722.1	agamous-like MADS-box protein AGL12 (LOC107421471)	Chr6	21238164–21257944+	699	232	26997.7	8.81	Type II	AGL12	8
*ZjMADS35*	XM_016035574.1	protein TRANSPARENT TESTA 16-like (LOC107425568)	Chr8	19035785–19038078−	753	250	29740.0	7.65	Type II	Bs/TT16	5
*ZjMADS36*	XM_016030485.1	MADS-box protein SVP-like (LOC107421277)	Chr6	15885156–15891994+	684	227	25660.5	6.37	Type II	SVP	7
*ZjMADS37*	XM_016035076.1	MADS-box protein JOINTLESS (LOC107425139), transcript variant X3	Chr8	12303276–12308153+	681	226	25613.8	5.74	Type II	SVP	7
*ZjMADS38*	XM_016038307.1	MADS-box protein JOINTLESS-like (LOC107427902), transcript variant X4	Chr9	24230013–24240337−	717	238	27397.54	6.48	Type II	SVP	7
*ZjMADS39*	XM_016029431.1	floral homeotic protein DEFICIENS-like (LOC107420465)	Chr6	5403081–5405702+	684	227	26479.3	9.37	Type II	AP3/PI	7
*ZjMADS40*	XM_016015291.1	floral homeotic protein PMADS 1-like (LOC107407949)	unplaced genomic scaffold, ZizJuj_1.1 add_scaffold377	1197–3569−	612	203	23613.7	9.43	Type II	AP3/PI	7
*ZjMADS41*	XM_016030269.1	floral homeotic protein PMADS 2 (LOC107421109)	Chr6	12081944–12084836−	573	190	22287.4	8.29	Type II	AP3/PI	6
*ZjMADS42*	XM_016042586.1	agamous-like MADS-box protein AGL18 (LOC107431619)	Chr12	1561435–1563861+	843	280	32106.0	7.10	Type II	AGL15	7
*ZjMADS43*	XM_016035038.1	MADS-box transcription factor 23 (LOC107425112)	Chr8	11798564–11804053−	678	225	25791.7	9.32	Type II	AGL17	7
*ZjMADS44*					663	220	25587.53	9.75	Type II	PI	3
*ZjMADS45*	XM_016026070.1	agamous-like MADS-box protein AGL15 (LOC107417456)	Chr5	367788–371560+	768	255	29071.1	6.68	Type II	AGL15	8
*ZjMADS46*	XM_016024828.1	agamous-like MADS-box protein AGL1 (LOC107416339)	Chr4	12872997–12878199+	735	244	28516.1	9.47	Type II	AG	6
*ZjMADS47*	XM_016038815.1	agamous-like MADS-box protein AGL9 homolog (LOC107428298)	Chr10	2910072–2914859−	738	245	27982.8	8.72	Type II	SEP	7
*ZjMADS48*	XM_016024921.1	developmental protein SEPALLATA 1 (LOC107416438)	Chr4	13375631–13379056−	735	244	27924.7	9.08	Type II	SEP	7
*ZjMADS49*	XM_016047608.1	agamous-like MADS-box protein AGL75 (LOC107435976)	unplaced genomic scaffold, ZizJuj_1.1 scaffold959	122769–123298−	387	128	14629.6	4.90	Type I	Mβ	2
*ZjMADS50*					624	207	23844.27	9.76	Type II	TM8	4
*ZjMADS51*	XM_016021625.1	agamous-like MADS-box protein AGL75 (LOC107413621)	Chr3	10258917–10259953−	948	315	35395.0	4.34	Type I	Mβ	1
*ZjMADS52*	XM_016017408.1	agamous-like MADS-box protein AGL62 (LOC107409993)	unplaced genomic scaffold, ZizJuj_1.1 add_scaffold819	834–1370−	537	178	20305.1	9.01	Type I	Mα	1

**Figure 1:**
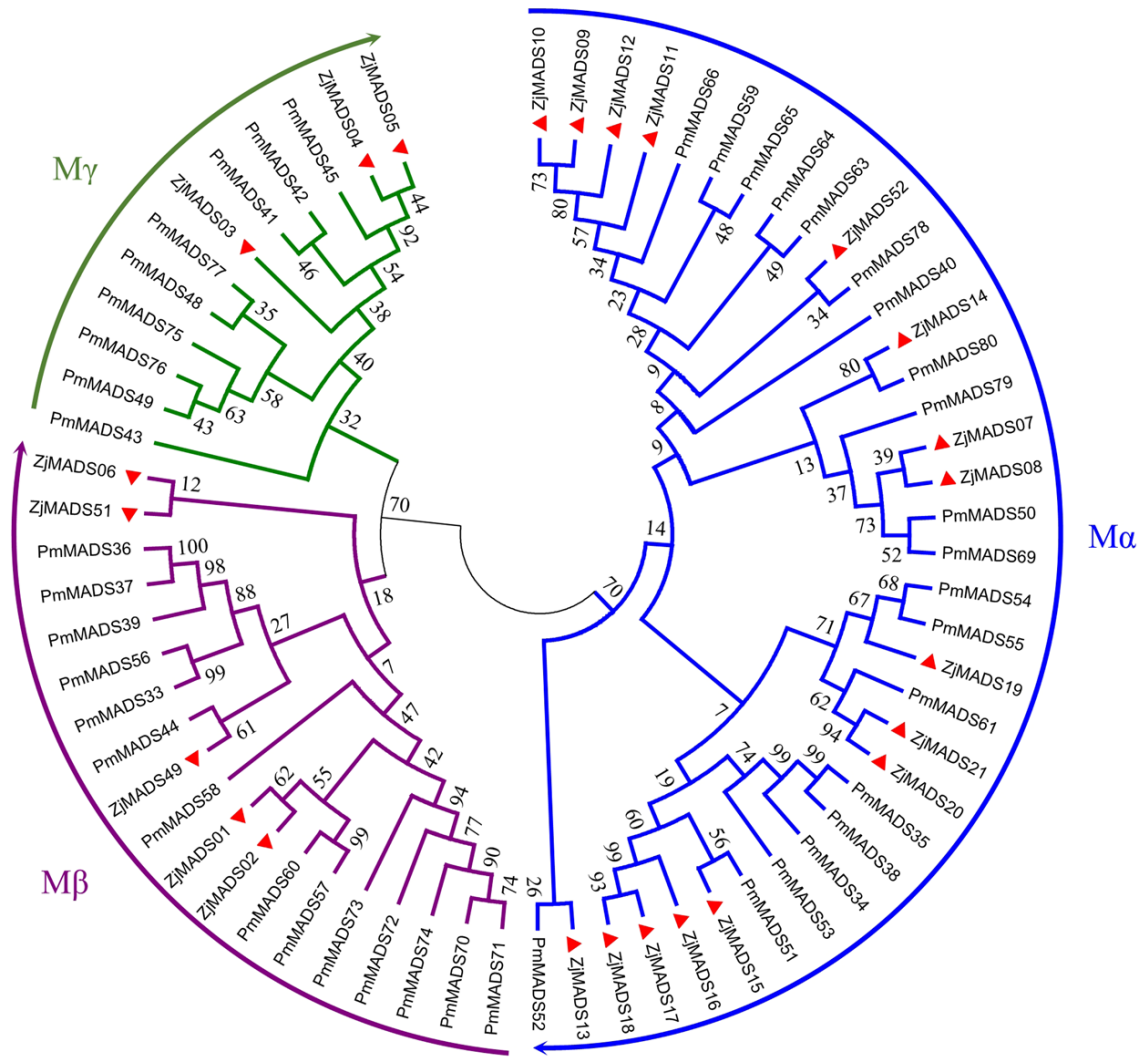
Phylogenetic tree of *Z*. *jujuba* and *P*. *mume* type I MADS-box genes. The phylogenetic tree was constructed based on the alignable region of protein sequence alignment of Chinese jujube MADS-box genes using the neighbor-joining method and no of differences model with bootstrapping analysis (1000 replicates). The subgroups are marked by different colors.

**Figure 2:**
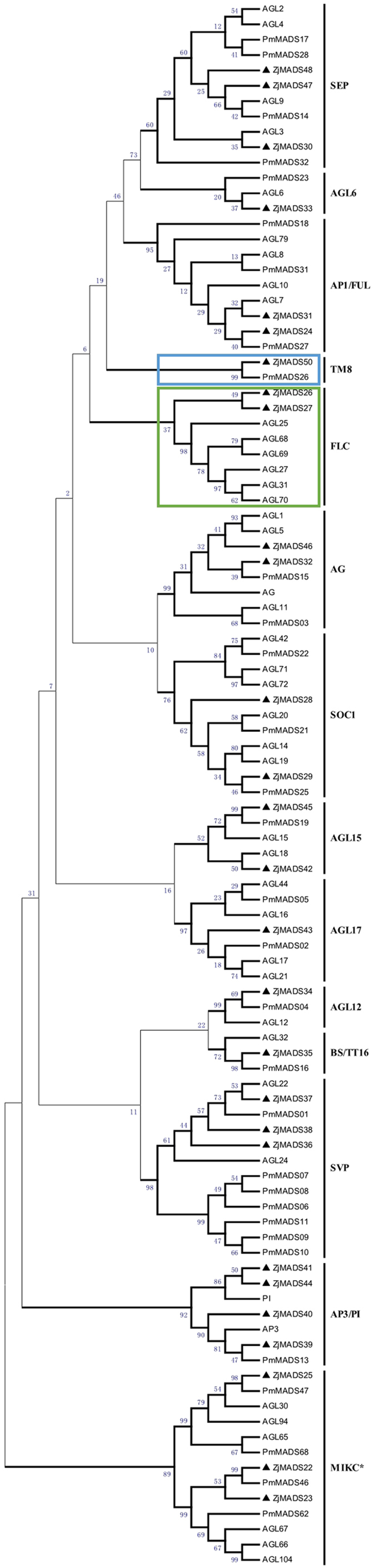
Phylogenetic tree of type II MADS-box genes in *Z*. *jujuba*, *P*. *mume* and *Arabidopsis*. The tree was generated using the neighbor-joining method implemented in MEGA 5.1. The FLC subfamily (absent in *P*. *mume*) and the TM8 subfamily (absent in *Arabidopsis*) are shown in frame sets.

Based on the phylogenetic analysis, 24 type I and 28 type II jujube genes were further divided into more detailed subgroups. Twenty-four type I genes were divided into Mα, Mβ, Mγ subgroups (Fig. 1) and, similar to *P*. *mume*, Mα was the group with the most genes. Sixteen out of 24 type I genes were classified into the Mα subgroup, while 5 were classified into the Mβ subgroup and 3 into the Mγ subgroup. Twenty-eight type II genes were further classified into 25 MIKC^C^-type and 3 MIKC*-type genes (Fig. 2). The 25 MIKC^C^-type genes were further divided into 13 clades: FLOWERING LOCUS C (FLC), SHORT VEGETATIVE PHASE (SVP), AGL6, TOMATO MADS-box 8 (TM8), AGL17, AGL15, AGL12, AG, AP3/PI, SUPPRESSOR OF OVEREXPRESSION OF CONSTANS 1 (SOC1), B-sister (BS/TT16), SEPALLATA (SEP), and APETALA1/FRUITFULL (AP1/FUL), each clade containing 1 to 4 MADS-box genes. In contrast to the *P*. *mume* genome, where an extreme expansion of the SVP clade was observed, phylogenetic analysis of the jujube type II genes showed that these genes were evenly distributed among different clades (Fig. 2).

### Gene structure and conserved motif distribution analysis

**Figure 3:**
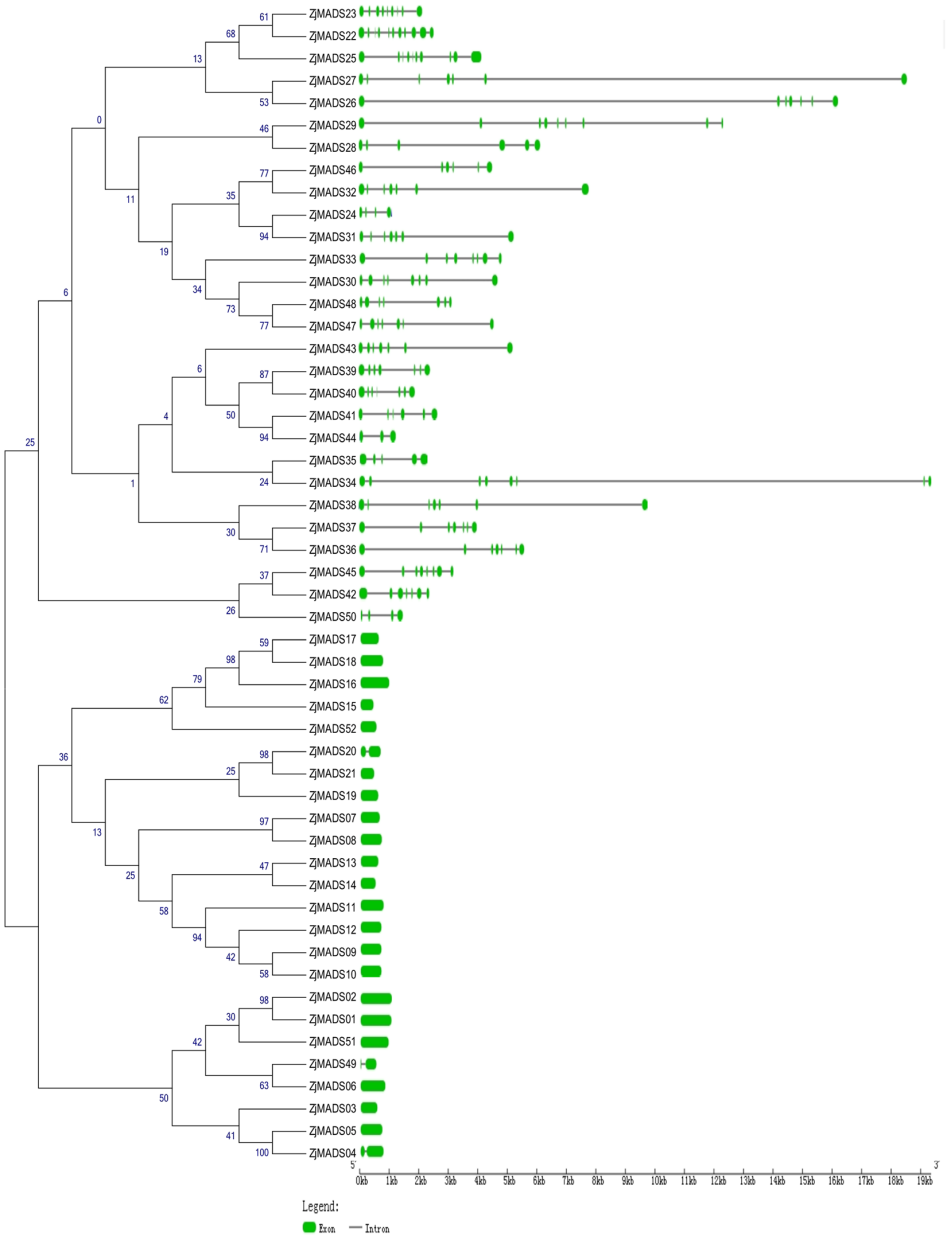
Phylogenetic relationships and structural analysis of MADS-box genes in the Chinese jujube. The unrooted neighbor-joining tree was constructed from the alignment of full-length amino acid sequences. Lengths of exons and introns of each MADS-box gene are displayed proportionally. Green solid boxes represent exons; black lines represent introns.

To better understand the structural diversity of MADS-box genes, we compared various intron/exon arrangements and conserved motifs based on their phylogenetic relationships. We obtained each gene structure by comparing their ORFs with their genomic sequences. As shown in Fig. 3, closely related genes were generally more structurally similar, differing only in intron and exon lengths. However, some close gene pairs exhibited distinct intron/exon arrangements. For example, *ZjMADS31* consisted of 7 exons, whereas its close paralog *ZjMADS24* had only 4 exons. In addition, we found that jujube MADS-box genes contained between 1 to 11 exons. The average number of exons in type II genes (7) was considerably greater than the average number of exons of the type I genes (1.12), which suggests that the type II MADS-box genes may be more complex.

We then used the MEME program to analyze conserved motifs in the MADS-box proteins, which had previously been subjected to SMART annotation. A total of 20 conserved motifs were identified (Supplementary Fig. S1). The commonly shared motifs tended to be in the same group: for instance, the SVP group included the *ZjMADS36*, *ZjMADS37* and *ZjMADS38* genes, all of which contain motifs 1, 2, 6, 7 and 9. Motif 1 was comprised of approximately 60 amino acids and was the most typical MADS-box domain. Motif 2 represented the K domain, which has been described to be the most conserved domain and essential for protein-protein interactions among MADS-box transcriptional factors. The K domain was identified in the majority of type II proteins, with one exception (*ZjMADS40*). This result is consistent with previous studies that showed that the K-box domain was only found in type II MADS-box genes^18^. However, in our study, we found that one type I gene, *ZjMADS51* also contained a K-box domain.

### Genomic distribution and duplication of jujube MADS-box genes

**Figure 4:**
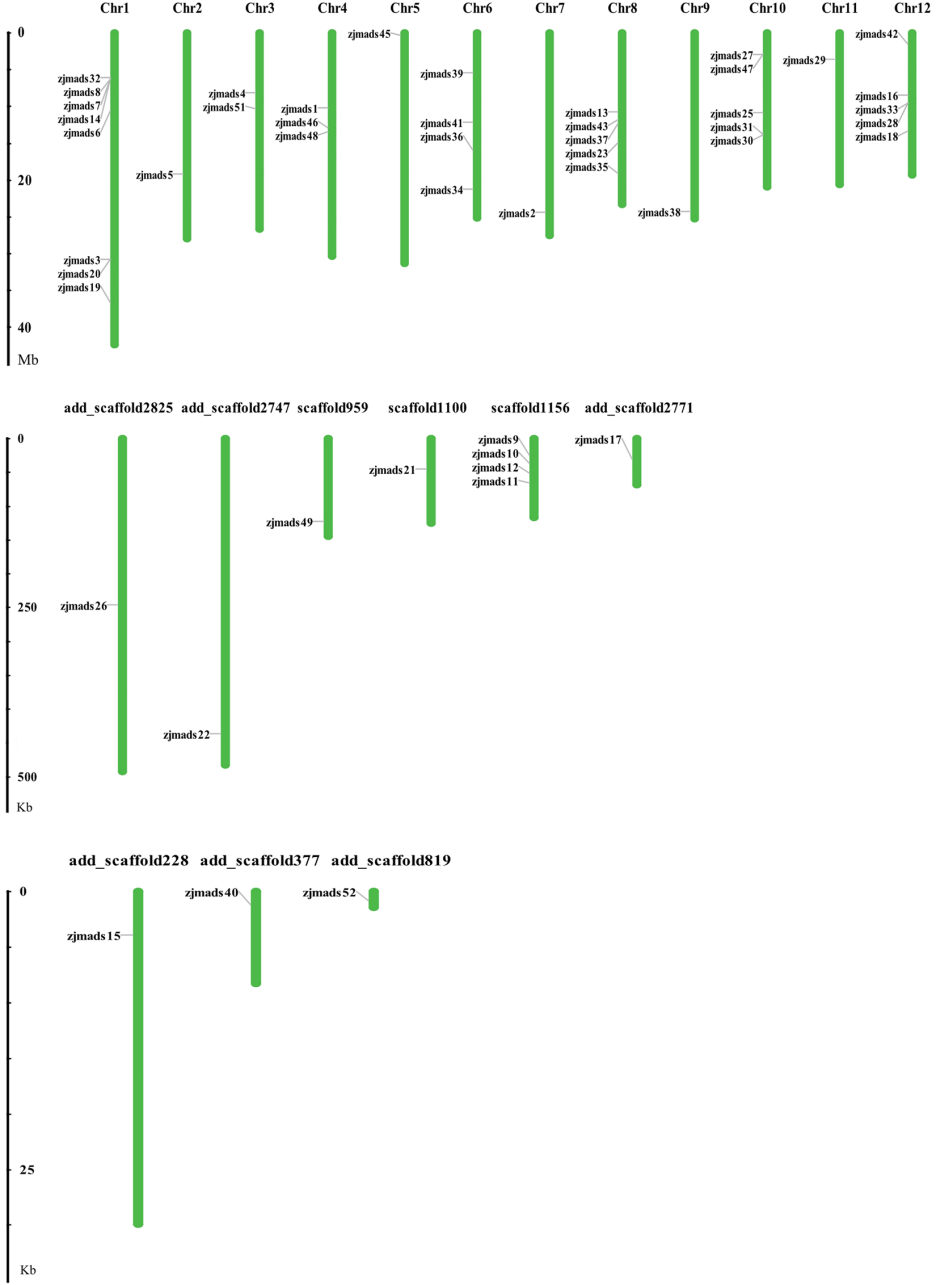
Positions of ZjMADS genes on the jujube pseudo-chromosomes or scaffolds.

We found that 37 of the 52 ZjMADS-box genes were randomly distributed across all 12 putative chromosomes, while 12 genes were assigned to unanchored scaffolds (Table 1 and Fig. 4). Three genes obtained by cloning were not anchored on chromosomes or scaffolds. Fourteen type I genes were mapped to 7 chromosomes, though most of the genes were located on chromosome 1. Twenty-three of the 28 type II genes were mapped to 9 chromosomes, and most of these were distributed on chromosomes 6, 8, 10 and 12. We also found clusters of some genes on chromosome 1, scaffold 1156 and chromosome 10 (*ZjMADS14*, *ZjMADS7* and *ZjMADS8*; *ZjMADS9*, *ZjMADS10* and *ZjMADS11*; *ZjMADS30* and *ZjMADS31*, respectively), and then we confirmed that tandem duplications have occurred in these genes. Further analyses should be performed to study the role of these duplications in the expansion of this family.

### Expression pattern of the jujube MADS-box genes in different tissues/organs

**Figure 5:**
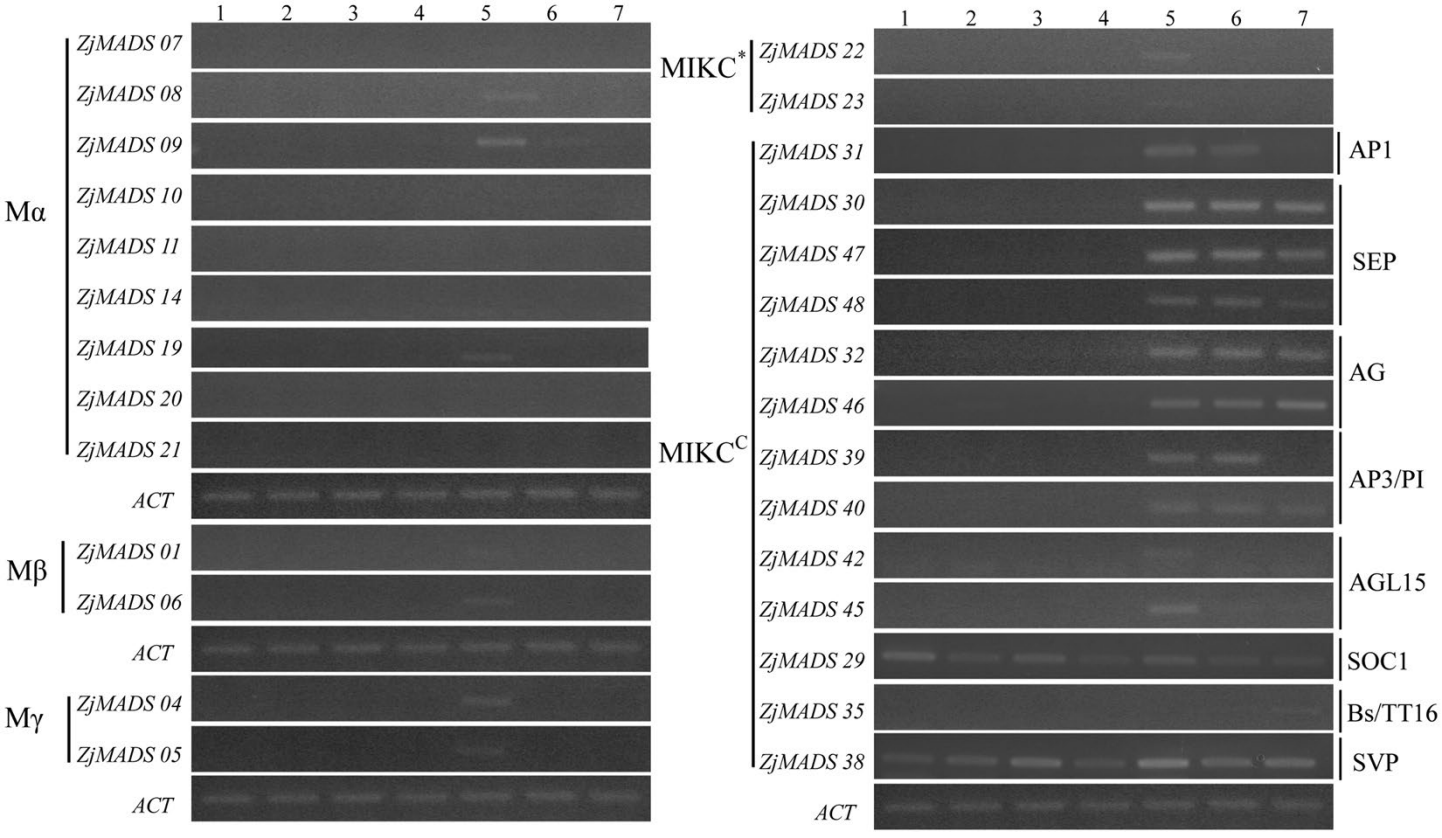
Expression patterns of jujube MADS-box genes in vegetative and reproductive organs by RT-PCR. Sources of the samples are as follow: 1-Root (R), 2-Young branch (YB), 3-Old branch (OB), 4-Leaf (L), 5-Flower Bud (B), 6-Flower (F) and 7-Young fruit (YF).

We investigated the expression patterns of 28 MADS-box genes in seven different tissues/organs (Fig. 5). Twenty-one MADS-box genes were expressed in at least one organ (especially in the flower bud), whereas the other 7 type I genes (*ZjMADS1*, *ZjMADS7*, *ZjMADS10*, *ZjMADS11*, *ZjMADS14*, *ZjMADS20* and *ZjMADS21*) were not detected in any of the organs tested. While most type I genes were only expressed in the flower bud, most type II genes were highly expressed in all reproductive tissues. Almost all of the MIKC^C^ genes exhibited the highest expressions in the flower bud and flowers, which further confirms their roles in jujube flower development.

Previous studies had shown that *SOC1* and its homologs in *Arabidopsis* were pivotal genes for flower transition and that *SOC1* overexpression could cause an early flowering phenotype^19, 20^. In our study, we found that *ZjMADS29* (the *SOC1* homolog) was highly expressed in the vegetative tissues. A high expression of *SOC1* in leaves was also found in *Arabidopsis* and tree peonies^21, 22^. In addition, we found that *ZjMADS38*, the *SVP* homolog, was widely expressed in various vegetative and propagation tissues/organs, which suggests that this gene may play multiple roles in jujube development.

### The critical MADS-box genes involved in jujube floral organ development

**Figure 6:**
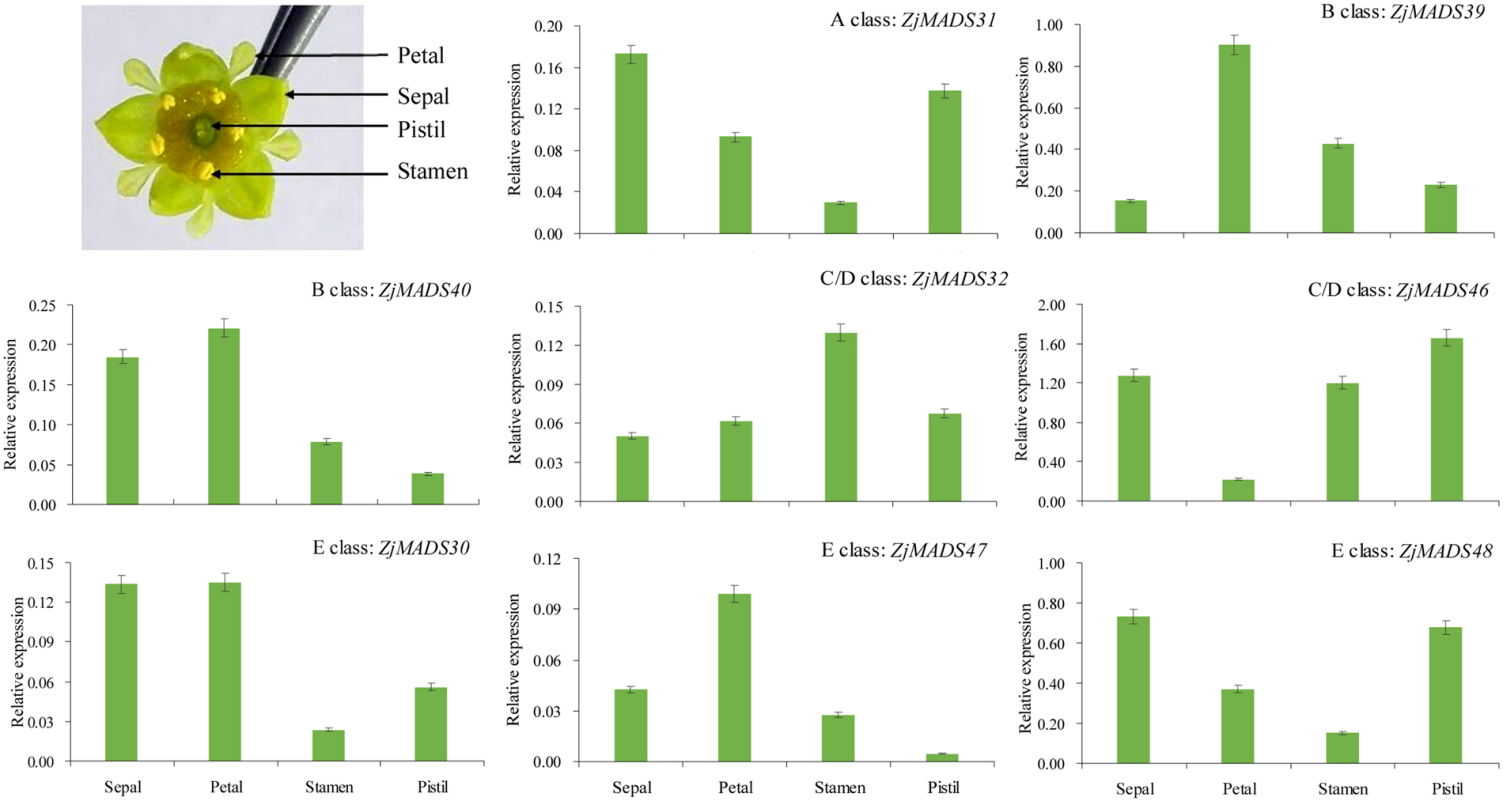
Expression patterns of 8 MIKC genes in four whorls of floral organs (sepal, petal, stamen and pistil) by RT-qPCR. *ZjACT* primers were used as the internal standard for each gene. The mean expression value was calculated from 3 independent replicates. The vertical bars indicate the standard deviation.

To further investigate the role of MADS-box genes in floral organ development, the expression patterns of 8 MIKC^C^ type genes were determined in four whorls (the sepal, petal, stamen and pistil) using a qRT-PCR assay. The expression of these 8 genes exhibited typical temporal and spatial expression patterns consistent with floral development (Fig. 6), suggesting a possible role in flower differentiation. The expression of *ZjMADS31* (an A-type gene) was notably high in the sepal; the expression of *ZjMADS39* and *ZjMADS40* (two B-type genes), was high in the petal but low in the pistil; transcripts of two C/D-type genes, *ZjMADS32* and *ZjMADS46*, were highly detected in the pistil and stamen; the expression of *ZjMADS30*, *ZjMADS47* and *ZjMADS48* (three E-type genes of the SEP subfamily), was high in the sepal, petal and pistil, indicating their overlapping expression profiles.

Phyllody caused by phytoplasma is one of typical symptoms in jujube trees infected by Jujube Witches’ Broom (JWB). The petal, stamen and pistil of phyllody in diseased jujube trees are the abnormal development of floral parts into sepal/leaf structures. The expression of *ZjMADS31* (A type gene) in flower and phyllody was higher than that in leaf (Supplementary Fig. S2), suggesting it played normal function in phyllody; however the increased expressions of B and C/D genes were observed in flower but not elevated in leaf and phyllody (Supplementary Fig. S2), indicating their function was nearly completely suppressed in phyllody and causing the abnormal development of petal, stamen and pistil; the expressions of three E-type genes in phyllody were higher than in leaf and lower than in flower (Supplementary Fig. S2), showing their function was partially inhibited in phyllody as compared to flower. The expressions of those genes were in agreement with above expression patterns in four whorls (Fig. 6). *ZjMADS47* is the homolog of Arabidopsis *SEP3* (NP_850953.1) in Chinese jujube and they shares 78% identity at the amino acid sequence level. Over-expression of *ZjMADS47* in Arabidopsis showed early flowering phenotype when compared with wild-type plants (Supplementary Fig. S3). RT-PCR analysis demonstrated that *AtSEP3* expression was up-regulated in transgenic lines. The results further highlighted that these genes were involved in jujube flower development.

## Discussions

MADS-box genes have been identified in several species, including *Arabidopsis* ^5^, poplar (*Populus trichocarpa*)^23^, grape (*Vitis vinifera* L.)^24^, apple (*Malus domestica*)^25^ and *P*. *mume* ^26^. In this study, we identified 52 non-redundant ZjMADS-box genes; this number may increase in the future when assembling or annotation problems in the jujube genome are addressed. Except for the tandem duplication, the segmental duplication should also contribute to the expansion of jujube MADS box genes. The difference in the number of MADS-box genes among the above-mentioned species (Supplementary Table S1) might be caused by a recent whole-genome duplication event. During the process of plant genome evolution, one or two whole-genome duplication events have previously been described in different species. According to previous studies, there has been one α and one β duplication event but no recent whole-genome duplication in Chinese jujube and *P*. *mume*, whereas two duplication events have occurred in apple. Thus, the number of apple MADS-box genes is greater than that of jujube and *P*. *mume*.

The average number of exons in the jujuba type II MADS-box genes was greater than that of the type I genes. Among the type II genes, 27 out of 28 (96.4%) contained more than 4 exons, while most type I genes had only one or two exons. The same phenomenon has also been observed in other species, such as *Arabidopsis* ^5^, apple^25^ and *P*. *mume* ^26^. The exon length distribution analysis also produced a similar pattern (Supplementary Table S2). These observations further highlight the conserved evolution among plants.

In contrast to type II MADS-box genes, information about type I genes is limited. Recent studies in *Arabidopsis* suggest that type I genes are important for plant reproduction and are required for proper development, especially for determining female gametophyte, and for proper embryo^27^ and endosperm development^28–30^. As in *Arabidopsis*, most type I genes in jujube were mainly expressed in flower buds or flowers, though the expression of some genes was too weak to detect. Compared to type I genes, most jujube type II genes were expressed in at least three organs, which suggests that these genes may have multiple functions in different organs. We found that gene pairs in the same clade can display similar or distinct expression profiles, which suggests possible functional redundancy or divergence, respectively. For instance, two *AP3*/*PI* genes (*ZjMADS39* and *ZjMADS40*) were highly expressed in the petal, implying that both genes may be able to perform B-related functions. In contrast, *ZjMADS40* was uniquely expressed in young fruits, whereas *ZjMADS39* was not, which indicates that these genes may have functionally diverged.

We believe that the expression profiles of MIKC^C^ type genes that we obtained in our study will be an important tool for elucidating flower development mechanisms in the Chinese jujube. Based on the bioinformatics analyses, the expression of the ABCDE genes in four whorls of jujube flower was very consistent with the predicted expression patterns, which suggests that they may indeed be involved in flower differentiation. As one of perennial woody plants, the genetic transformation system of Chinese jujube was still not established, therefore it is very difficult to perform functional study in jujube. Phyllody as the suitable tissue for studying jujube flower development was applied in this study (Supplementary Fig. S2). The expression pattern of A type gene in phyllody was similar to flower, suggesting A type gene should play the normal function in phyllody. However the low level expression of B, C/D and E type genes in phyllody indicated that their function were remarkable inhibited, causing the abnormal development of petal, stamen and pistil. We demonstrated that over-expressing of *ZjMADS47* (homolog with *SEP3* in Arabidopsis) caused early flowering in *Arabidopsis* plants, which is in accordence with the *SEP3* function in Arabidopsis and lily^31, 32^. This result was supported the functional conservation of MADS-box genes between jujube and other plants.

In addition, *ZjMADS46* is a SHATTERPROOF-like gene that showed high similarity to *Prunus persica* (85%), *Prunus triloba* (85%) and *Fragaria x ananassa* (81%) genes. SHATTERPROOF-like genes have been shown to regulate fruit ripening in Arabidopsis^33^, peach^34^, strawberry^35^, and oil palm^36^. In our study, *ZjMADS46* exhibited the highest expression in the pistil (Fig. 6); more studies are needed to further investigate the role of *ZjMADS46* in jujube fruit development.

In summary, our results have laid the foundation for a thorough functional characterization of the MADS-box gene family in jujube and have hopefully enabled a better understanding of the structure-function relationship between MADS-box gene family members. Additionally, our study provides comprehensive information and novel insights into the evolution and divergence of the MADS-box genes in plants. Finally, studies similar to this may potentially aid in the understanding of the molecular basis of many agriculturally important jujube traits such as flower and fruit development and other physiological processes.

## Materials and Methods

### Plant material

Roots (R), young branches (YB), old branches (OB), leaves (L), flower buds (B), flowers (F) and young fruits (YF) were collected from three jujube trees. Four different floral organs (the sepal, petal, stamen and pistil) were harvested at florescence time (May 29, 2016). The Chinese jujube flowers are very small (approximately 5 mm). Four whorls were quickly separated and frozen in liquid nitrogen in the field. Phyllody is one of typical symptoms in jujube trees infected by JWB disease. Three tissues, i.e. leaf, phyllody and flower, were applied to study the expression of genes related to floral development. Three biological replicates were collected in each treatment. The samples were stored at −80 °C for RNA extraction and expression analysis.

### Identification of MADS-box genes

To identify MADS-box genes in the jujube genome, previously identified *Arabidopsis* MADS-box sequences were submitted to the Pfam database (http://pfam.sanger.ac.uk)^37^ to obtain the domain architecture of this family. The hidden Markov model (HMM) profiles of the SFR (type I) domain (PF00319) and the Myocyte Enhancer Factor-2 MEF2 (type II) domain (PF09047) were retrieved from Pfam^38^. MADS-box genes were then identified in the jujube genome database^39^ using the hidden Markov model (HMM) profile corresponding to the Pfam MADS-box family PF00319 and PF09047 domains, using the HMMER version 3.0 software^40^. Finally, we further verified these sequences using the SMART tool (http://smart.embl-heidelberg.de/)^41^ combined with the Pfam (http://Pfam.sanger.ac.uk/) and the NCBI databases (http://www.ncbi.nlm.nih.gov/). The sequences lacking MADS domains were rejected in this analysis. A total of 49 MADS-box proteins were obtained and used for further analysis. 3 MADS-box proteins obtained from homologous cloning were also included in this study. The online tool ProtParam (http://web.expasy.org/protparam/) was employed to predict the molecular weight and isoelectric point (pI) of each protein.

### Phylogenetic analysis

The *Arabidopsis thaliana* MADS proteins were retrieved from the TAIR database (http://www.arabidopsis.org/) based on a previous report^5^. The *Prunus mume* genome sequences were downloaded from the *P*. *mume* genome project website (http://prunusmumegenome.bjfu.edu.cn/), and the dataset of the predicted *P*. *mume* MADS proteins was retrieved from previous analyses^26^. Following the convention established for *P*. *mume* and Arabidopsis MADS proteins, the jujube MADS proteins were classified into different groups. Multiple sequence alignments were performed using Clustal X2.0 with default parameters^42^. A phylogenetic tree was then constructed using the neighbor-joining method, and bootstrap values were calculated with 1,000 replications using MEGA5.1^43^.

### Conserved motif and gene structure analysis

To identify the conserved motifs in the Chinese jujube full-length MADS proteins, the Multiple Expectation-maximization for Motif Elicitation (MEME) program version 4.9.0^44^ was used with mostly default parameters, except for the following: (1) the optimum motif width was set to ≥6 and ≤60 and (2) the maximum number of motifs was set to 20. The MEME motifs were then annotated using the SMART program (http://smart.embl-heidelberg.de) and the Pfam database.

The coding domain sequences (CDS) and DNA sequences of the MADS-box genes were used to predict gene structure using the online tool GSDS^45^ (http://gsds.cbi.pku.edu.cn), which allowed us to infer both exon position and gene length. Then, exon lengths of the jujube MIKC^C^ genes were estimated and compared with those of *Arabidopsis* and apple genes.

### Chromosomal location and gene duplication

To determine the chromosomal location of the MADS-box genes, the MADS-box gene sequences were further used as query sequences in BLASTN searches against the jujube genome sequence. Each MADS-box gene was thus mapped to the jujube genome according to their coordinates on the genome. Tandem duplications were identified according to previously described methods^46^.

### RNA isolation and expression analysis

Total RNA was isolated from 100 mg of frozen tissue using an RNA kit (RNAprep Pure Plant Kit, Tiangen, Beijing, China) according to the manufacturer’s instructions. First-strand cDNA was synthesized from 2 μg of RNA using the TIANScript First Strand cDNA Synthesis Kit (Tiangen, Beijing, China) according to manufacturer’s instructions. The resulting cDNA was then diluted nine-fold and stored at −20 °C for the subsequent semi-quantitative RT-PCR and qRT-PCR assays.

For gene expression quantification, specific primers were designed for each MADS-box gene using the Primer Premier 5.0 software and expression patterns were assayed by semi-quantitative RT-PCR. PCR reactions were performed using the following program: initial denaturation at 95 °C for 3 min; 30 cycles at 95 °C for 15 s, 55 °C for 15 s, and 72 °C for 30 s, and a final extension cycle at 72 °C for 10 min. *ZjACT* was used as reference gene^45^. RT-PCR products were then sequenced to ensure that they were derived from the desired target genes. Primer details primers are listed in the Supplementary Table S3.

The expression of 8 MADS-box genes in different floral organs was examined using qRT-PCR. Total RNA was extracted from the sepal, petal, stamen and pistil. qRT-PCR was performed using the Bio-Rad iQ5 detection system. Reactions were performed in a 20 μL volume containing 1 μL of cDNA, 400 nM of each primer and 10 μL of SYBR Green mix, according to the TransStart Top Green qPCR SuperMix instructions. The reactions were performed under the following conditions: 94 °C for 30 s, and 40 cycles of 94 °C for 5 s, 55 °C for 15 s and 72 °C for 15 s. The specificity of the amplicon for each primer pair was verified by melting curve analysis. All the experiments were performed in three biological replicates, and each replicate was measured in triplicate. The relative expression levels were calculated using the 2^−ΔCt^ method and with *ZjACT* as the reference gene^47^.

## Electronic supplementary material

Supplementary Materials
