# Supplementary material for: Genome-wide identification, characterization of the MADS-box gene family in Chinese jujube and their involvement in flower development
Source: Sci Rep. 2017 Apr 21;7:1025. doi: 10.1038/s41598-017-01159-8 (PMC5430891; doi:10.1038/s41598-017-01159-8)
Supplement: Supplementary file 1 — Supplementary Materials [file 41598_2017_1159_MOESM1_ESM.pdf]

Genome-wide identification, characterization of the MADS-box gene family in Chinese jujube and their involvement in flower development

Liman Zhang [1], Jin Zhao [1] ✉, Chunfang Feng [2], Mengjun Liu[3] ✉, Jiurui Wang [2] & Yafei Hu [4]

[1] College of Life Science, Hebei Agricultural University, Baoding 071000, China. [2] College of Forestry, Hebei Agricultural University, Baoding 071000, China. [3] Research Center of Chinese Jujube, Hebei Agricultural University, Baoding 071000, China. [4] BGI-Shenzhen, Shenzhen 518083, China. Correspondence and requests for materials should be addressed to J. Z. (email: zhaojinbd@126.com) or M. L.(email: lmj1234567@aliyun.com)

Supplementary Materials

Supplementary Figure S1 Conserved motif analyses of MADS-box proteins in Chinese jujube. The genes are on the left of the motif bars and the phylogenetic relationship is shown. Each motif is represented by a number in a colored box. Box length corresponds to motif length.

Supplementary Figure S2 Expression patterns of 8 MIKC genes in leaf, phyllody and flower by qRT-PCR. *ZjACT* primers were used as the internal standard for each gene. The mean expression value was calculated from 3 independent replicates. The vertical bars indicate the standard deviation.

Supplementary Figure S3 Over-expressing (OX) of *ZjMADS47* causes early flowering in *Arabidopsis* plants. *Arabidopsis thaliana* ecotype Col-0 was used as the WT. (A) More roots were observed in *ZjMADS47*-OX *Arabidopsis* plants. (B) The early flowering phenotype was observed in *ZjMADS47*-OX *Arabidopsis* plants. (C) *ZjMADS47* and *AtSEP3* expressions in WT and OX plants were analyzed by RT-PCR using the *ACT* gene as a reference. *Arabidopsis* plants were grown under 16 h LD at 23-25°C.

Figure 5. Expression patterns of jujube MADS-box genes in vegetative and reproductive organs by RT-PCR. Sources of the samples are as follow: 1-Root (R), 2-Young branch (YB), 3-Old branch (OB), 4-Leaf (L), 5-Flower Bud (B), 6-Flower (F) and 7-Young fruit (YF).

Supplementary Table S1 The number of MADS-box genes in *Arabidopsis*, poplar, grape, *Prunus mume*, apple and jujube.

Supplementary Table S2 Exon length distribution analysis of MIKC genes in *Arabidopsis*, apple and jujube

Supplementary Table S3 The primer information of *ZjMADSs* used in this study

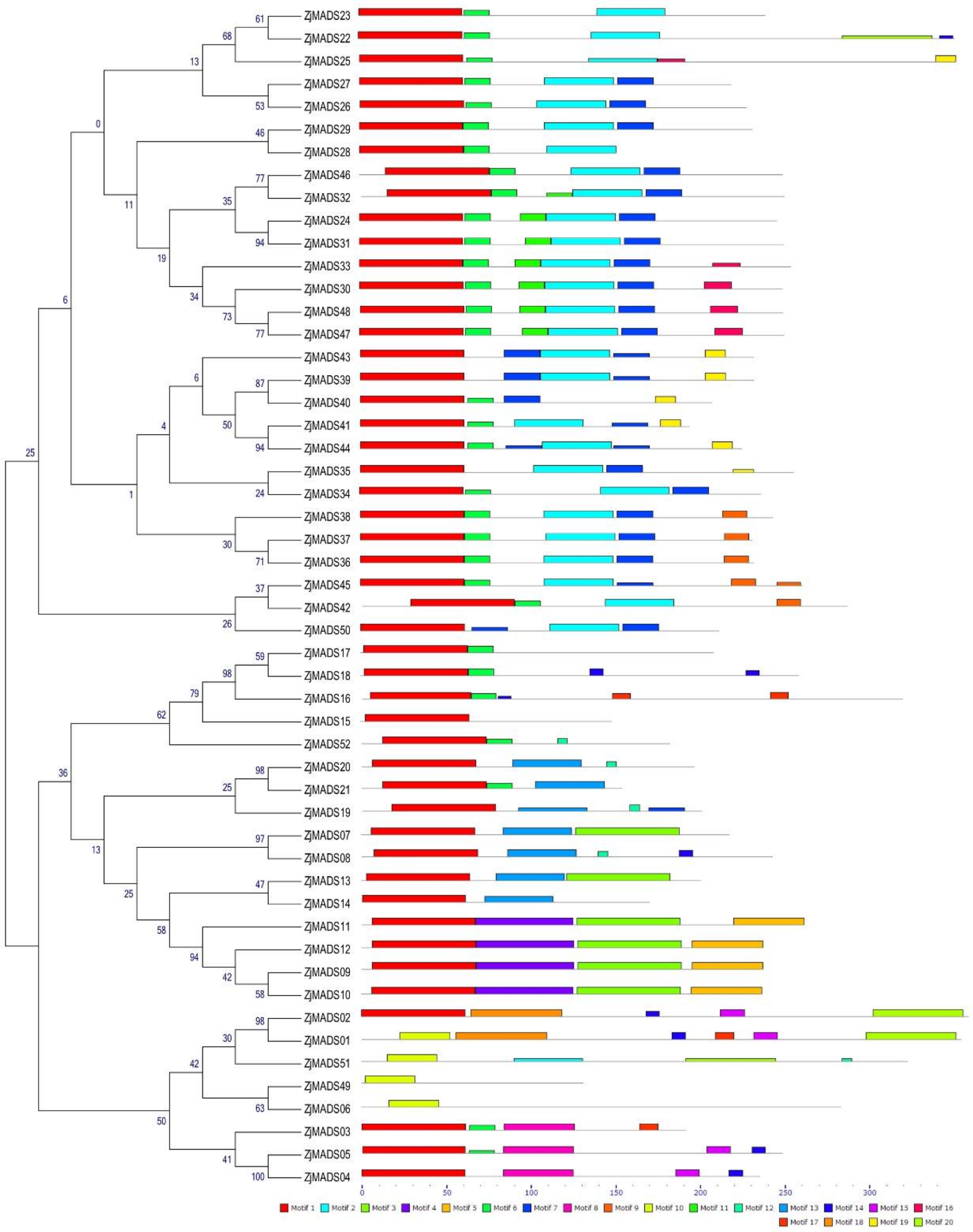

Supplementary Figure S1 Conserved motif analyses of MADS-box proteins in Chinese jujube. The genes are on the left of the motif bars and the phylogenetic relationship is shown. Each motif is represented by a number in a colored box. Box length corresponds to motif length.

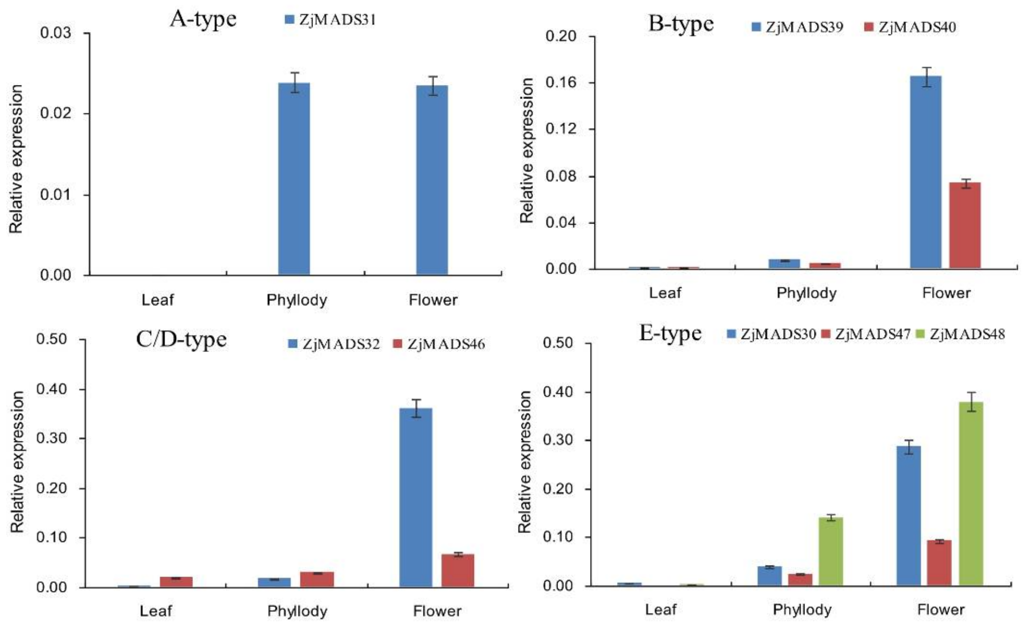

Supplementary Figure S2 Expression patterns of 8 MIKC genes in leaf, phyllody and flower by qRT-PCR. *ZjACT* primers were used as the internal standard for each gene. The mean expression value was calculated from 3 independent replicates. The vertical bars indicate the standard deviation.

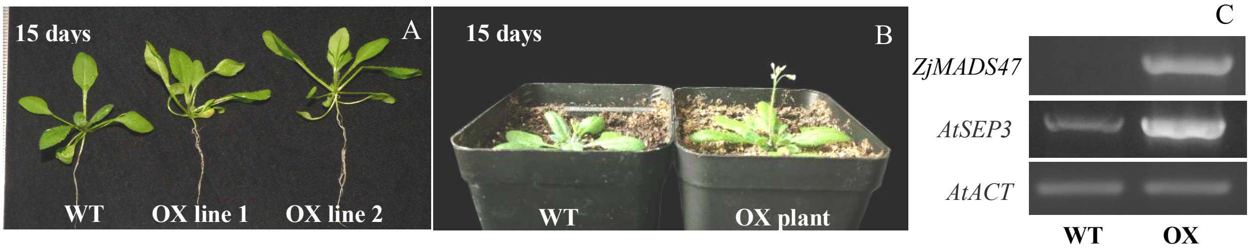

Supplementary Figure S3 Over-expressing (OX) of *ZjMADS47* causes early flowering in *Arabidopsis* plants. *Arabidopsis thaliana* ecotype Col-0 was used as the WT. (A) More roots were observed in *ZjMADS47*-OX *Arabidopsis* plants. (B) The early flowering phenotype was observed in *ZjMADS47*-OX *Arabidopsis* plants. (C) *ZjMADS47* and *AtSEP3* expressions in WT and OX plants were analyzed by RT-PCR using the *ACT* gene as a reference. *Arabidopsis* plants were grown under 16 h LD at 23-25°C.

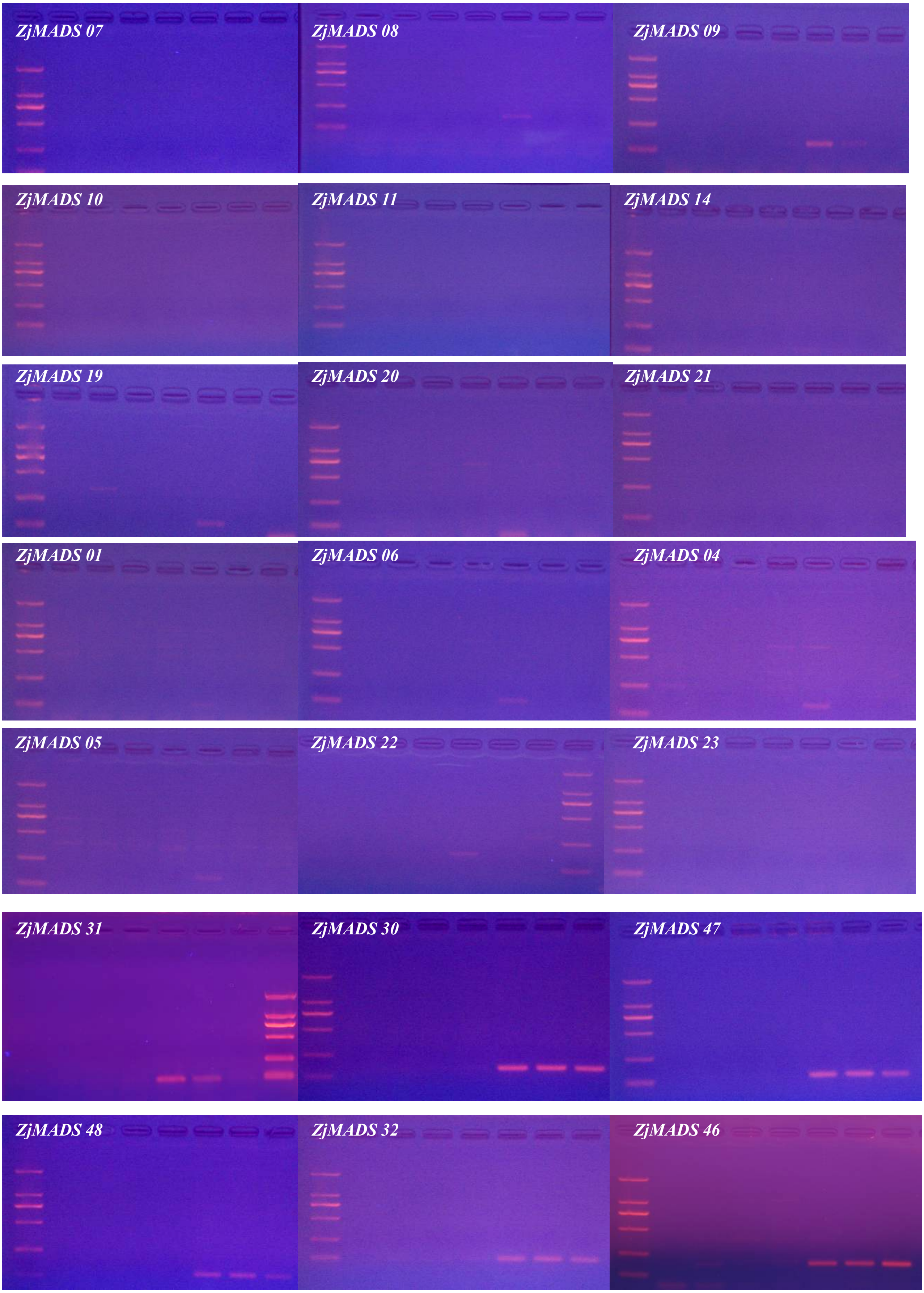
ZjMADS 07
ZjMADS 08
ZjMADS 09
ZjMADS 10
ZjMADS 11
ZjMADS 14
ZjMADS 19
ZjMADS 20
ZjMADS 21
ZjMADS 01
ZjMADS 06
ZjMADS 04
ZjMADS 05
ZjMADS 22
ZjMADS 23
ZjMADS 31
ZjMADS 30
ZjMADS 47
ZjMADS 48
ZjMADS 32
ZjMADS 46

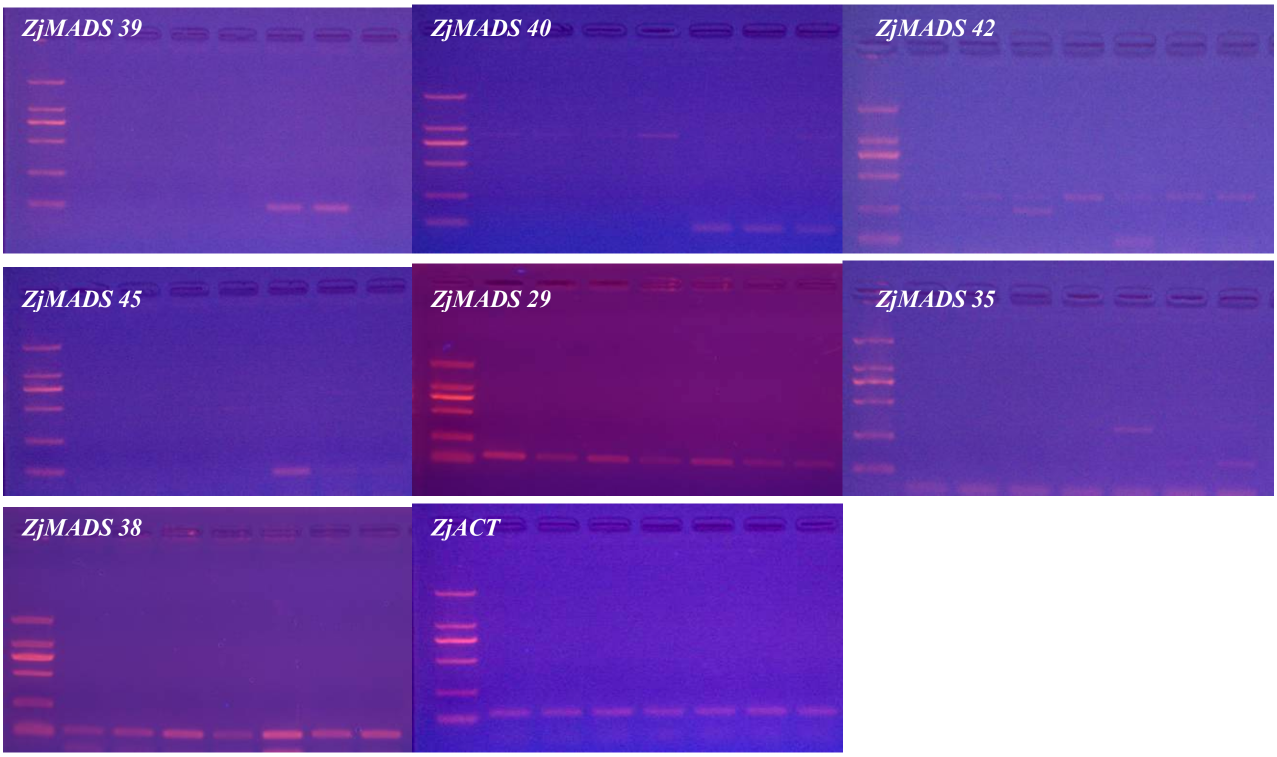

Figure 5. Expression patterns of jujube MADS-box genes in vegetative and reproductive organs by RT-PCR. Sources of the samples are as follow: 1-Root (R), 2-Young branch (YB), 3-Old branch (OB), 4-Leaf (L), 5-Flower Bud (B), 6-Flower (F) and 7-Young fruit (YF)

Table S1 The number of MADS-box genes in Arabidopsis, poplar, grape, *Prunus mume,* apple and jujube.

Species	MIKC^C	MIKC*	Type II	Mα	Mβ	Mγ	Mδ	Type I	Total
Arabidopsis thaliana (Parenicova et al., 2003)	43	2	45	20	17	21	4	62	107
Populus trichocarpa (Leseberg et al., 2006)	55	2	57	23	12	6	7	48	105
Vitis vinifera (Diaz-Riquelme et al., 2009)	32	-	-	-	-	-	-	-	-
Prunus mume (Xu et al., 2014)	-	-	32	20	14	9	5	48	80
Malus domestrica (Velasco et al., 2010)	75	7	82	22	8	24	10	64	146
Ziziphus jujuba	25	3	28	16	5	3	-	24	52

Table S2 Exon length distribution analysis of MIKC genes in Arabidopsis, apple and jujube

Species	Exon length (bp)					
	182/185	79/82	62	100	42	42
Arabidopsis thaliana	85.70%	65.70%	74.20%	91.40%	94.20%	82.90%
Malus domestrica	54.90%	28%	45.10%	47.60%	39%	30.50%
Ziziphus jujuba	60%	40%	56%	76%	76%	56%

Note: (1) the length of two exons were both 42bp. (2) *ZjMADS24, 44, 50* excluded.

Table S3 The primer information of *ZjMADSs* used in this study

Gene name	Primers (5'-3')	Tm (°C)	Size (bp)
ZjMADS01	CTCCTCAAATGGTTCCTCCT<br>ATCGTTCATCAGCATCATCA	60	101
ZjMADS04	AGCGAGCTGAGTACCCTTTG<br>CTTGTTCTGCTCCATTGCCG	64	147
ZjMADS05	TGAAGAAGGTGGGCGAGTTG<br>CCATCTCCGGCATCTTCTTG	63	147
ZjMADS06	GCATCGCACTCACTTTCC<br>TCAGTAGTCTCCAGATC	56.9	94
ZjMADS07	GAGAATGAAGATGATCGCCT<br>ACCGGTTCGCTATAGACTCAAT	60	172
ZjMADS08	TCTCCAAACGCAGATCTGG<br>CATTGGTGTCTTGAAGCG	58	177
ZjMADS09	GGGCCATGAACTGAATCGGA<br>CGTGCTTGCTCACGTTCTTC	64	140
ZjMADS10	GGGGCGGCGATGATTACTT<br>TGGTGATGATGAGGC	58.1	89
ZjMADS11	TCCAGCACGATGCCACTC<br>GCTTCTTCTCCGCCTCCA	59	100
ZjMADS14	GGATAGCAGTTCCAGGCAGG<br>CCACCTGGTGAGAAGACGAC	59	120
ZjMADS19	AGGCTGTACCGCTGACAATC<br>CTGATCACCGCACCTTGTCT	60	121
ZjMADS20	TTCTAATTCCTCAGAGGCGGC<br>CACTCCACGCCACCTTCTTT	59	188
ZjMADS21	CGCTACATTCACAGCACCAC<br>CACGGCAAGTTTCACCTCCT	59.7	118
ZjMADS22	TGGGAAGAGTGAAGCTCCAAAT<br>TTGCCGGAGAAGAGACTGAC	59	178
ZjMADS23	ATCAGGCCGTCTCAGTCACTT<br>GAAGCTCATGCTGCAAACTG	59	248
ZjMADS29	GGAAAAACTCAGATGAAG<br>GGAGAGAAAATTATAAGGGCAAC	55	140
ZjMADS35	AACAGTATGTGCCGTTGGGT<br>GTTGCAAGCTGAAGCACACT	61	143
ZjMADS38	TCCAGATCAAGAAGATCGAC<br>GAGCAATCTCAGCATCACAGAG	55	177
ZjMADS42	TCGCTAGCAACAGCATGGAA<br>CTATGGTCAGGACGACGCTC	61	97
ZjMADS45	TACCACATTGCACTTGGGGC<br>CCAATTGGCTTCCCGAGTCA	61	95
ZjMADS30	GGGGAGAGGTAAGGTGGAG<br>GGTTGGAGAAGATGATAAGAGC	58	149

ZjMADS31	CTCTGCTTCGTCATCCTTCC	58	91
	CCTCATTTCTGGGGCTTCT		
ZjMADS32	TAGCAGAGGACGCCTTT	52	194
	CACCCAGCATCTCCCTATTT		
ZjMADS39	GATTAGTTGACAATAGAGGGG	55	109
	CTGTGAAGGTTAGGCTGG		
ZjMADS40	TTGAGAACCACACCAACAGG	59	93
	TGGCATCGCATAGAACAGT		
ZjMADS46	CGGCCGCCTCTATGAGTATG	59	130
	GGCCTCCTGCTGGTAAAACT		
ZjMADS47	GGGGAAGACCTTGGACCTTT	59	143
	AGCAAGTGTTCCTTGCGTTG		
ZjMADS48	GGGGTTCTTCCAGGCCTTAG	60	99
	ACTTGCTGGGCATGACTTGT		
